# Supplementary material for: Predominant Gram-Positive Etiology May Be Associated with a Lower Mortality Rate but with Higher Antibiotic Resistance in Spontaneous Bacterial Peritonitis: A 7-Year Study in a Tertiary Center in Romania
Source: Life (Basel). 2025 May 26;15(6):855. doi: 10.3390/life15060855 (PMC12194169; doi:10.3390/life15060855)
Supplement: Supplementary file 1 [file life-15-00855-s001.zip › life-3642854-supplementary.pdf]

Table S1 Antibiotic resistance in non-nosocomial and nosocomial ascitic fluid infection

	Non-nosocomial Resistance/Total (%)	Nosocomial Resistance/Total (%)	TOTAL Resistance/Total (%)
Amikacin	4/13 (30.8)	8/14 (57.1)	12/27 (44.4)
Aztreonam	0/7 (0)	0/7 (0)	0/14 (0)
Ampicillin	7/13 (53.8)	7/8 (87.5)	14/21 (66.7)
Ampicillin + Clavulanic	3/8 (37.5)	3/7 (42.9)	6/15 (40)
Ampicillin + Sulbactam	4/6 (66.7)	4/7 (57.1)	8/13 (61.5)
Cefazolin	4/6 (66.7)	5/6 (83.3)	9/12 (75)
Cefepime	1/11 (9.1)	5/12 (41.7)	6/23 (26.1)
Cefotaxime	1/7 (14.3)	6/8 (75)	7/15 (46.7)
Cefoxitin	3/8 (37.5)	4/6 (66.7)	7/14 (50)
Ceftazidime	2/7 (28.6)	8/12 (66.7)	10/19 (52.6)
Cefoperazone+ Sulbactam	0/3 (0)	1/3 (33.3)	1/6 (16.7)
Ceftazidime + Avibactam	0/2 (0)	0/2 (0)	0/4 (0)
Ceftaroline	1/2 (50)	3/4 (75)	4/6 (66.7)
Ceftriaxone	3/10 (30)	5/10 (50)	8/20 (40)
Cefuroxime	2/6 (33.3)	4/7 (57.1)	6/13 (46.2)
Ciprofloxacin	7/25 (28)	14/28 (50)	21/53 (39.6)
Clarithromycin	7/17 (41.2)	7/12 (58.3)	14/29 (48.3)
Clindamycin	8/18 (44.4)	7/13 (53.8)	15/31 (48.4)
Chloramphenicol	5/24 (20.8)	2/16 (12.5)	7/40 (17.5)
Colistin	1/6 (16.7)	0/8 (0)	1/14 (7.1)
Levofloxacin	3/15 (20)	6/16 (37.5)	9/31 (29)
Norfloxacin	0/1 (0)	1/1 (100)	1/2 (50)
Ofloxacin	2/5 (40)	1/4 (25)	3/9 (33.3)
Moxifloxacin	2/15 (13.3)	1/11 (9.1)	3/26 (11.5)
Doxycycline	4/15 (26.7)	3/14 (21.4)	7/29 (24.1)
Nitrofurantoin	0/2 (0)	1/3 (33.3)	1/5 (20)
Quinupristin	0/1 (0)	-	0/1 (0)
Linezolid	0/19 (0)	0/16 (0)	0/35 (0)
Erythromycin	9/17 (52.9)	8/13 (61.5)	17/30 (56.7)
Gentamycin	4/15 (26.7)	9/23 (39.1)	13/38 (34.2)
Ertapenem	0/5 (0)	2/4 (50)	2/9 (22.2)
Imipenem	0/7 (0)	5/10 (50)	5/17 (29.4)
Meropenem	0/9 (0)	7/11 (63.6)	7/20 (35)
Oxacillin	12/18 (66.7)	8/13 (61.5)	20/31 (64.5)
Penicillin	15/21 (71.4)	16/18 (88.9)	31/39 (79.5)
Piperacillin	1/1 (100)	3/3 (100)	4/4 (100)
Rifampicin	3/14 (21.4)	3/11 (27.3)	6/25 (24)
TMP+SMTX	8/20 (40)	11/22 (50)	19/42 (45.2)
Piperacillin + Tazobactam	0/6 (0)	6/9 (66.7)	6/15 (40)
Teicoplanin	1/2 (50)	0/2 (0)	1/4 (25)
Tetracycline	10/21 (47.6)	7/22 (31.8)	17/43 (39.5)
Tigecycline	2/15 (13.3)	3/14 (21.4)	5/29 (17.2)
Tobramycin	2/4 (50)	4/7 (57.1)	6/11 (54.5)
Vancomycin	0/6 (0)	0/7 (0)	0/13 (0)

Table S2. *In vitro* resistance to antibiotics for Gram-positive and Gram-negative ascitic infection						
	G-POSITIVE Resistance/Total (%)			G-NEGATIVE Resistance/Total (%)		
	Non-N	N	ALL	Non-N	N	ALL
Amikacin	3/8 (37.5)	3/4 (75)	6/12 (50)	1/5 (20)	5/10 (50)	6/15 (40)
Aztreonam	-	-	-	0/7 (0)	0/7 (0)	0/14 (0)
Ampicillin	1/7 (14.3)	3/4 (75)	4/11 (36.4)	6/6 (100)	4/4 (100)	10/10 (100)
Ampicillin + Clavulanic	0/1 (0)	-	0/1 (0)	3/7 (42.9)	3/7 (42.9)	6/14 (42.9)
Ampicillin + Sulbactam	-	-	-	4/6 (66.7)	4/7 (57.2)	8/13 (61.5)
Cefazolin	-	-	-	4/6 (66.7)	5/6 (83.3)	9/12 (75)
Cefepime	0/3 (0)	0/2 (0)	0/5 (0)	1/8 (12.5)	5/10 (50)	6/18 (33.3)
Cefotaxime	0/3 (0)	0/2 (0)	0/5 (0)	1/4 (25)	6/6 (100)	7/10 (70)
Cefoxitin	1/1 (100)	-	1/1 (100)	2/7 (28.6)	4/6 (66.7)	6/13 (46.2)
Ceftazidime	-	-	-	2/7 (28.6)	8/12 (66.7)	10/19 (52.6)
Cefoperazone+ Sulbactam	-	-	-	0/3 (0)	1/3 (33.3)	1/6 (16.7)
Ceftazidime + Avibactam	-	-	-	0/2 (0)	0/2 (0)	0/4 (0)
Ceftaroline	1/2 (50)	2/3 (66.7)	3/5 (60)	-	1/1 (100)	1/1 (100)
Ceftriaxone	1/2 (50)	0/2 (0)	1/4 (25)	2/8 (25)	5/8 (62.5)	7/16 (43.75)
Cefuroxime	-	-	-	2/6 (33.3)	4/7 (57.1)	6/13 (46.2)
Ciprofloxacin	4/17 (23.5)	6/16 (37.5)	10/33 (30.3)	3/8 (37.5)	8/12 (66.7)	11/20 (55)
Clarithromycin	7/17 (41.2)	7/12 (58.3)	14/29 (48.3)	-	-	-
Clindamycin	8/18 (44.4)	7/13 (53.8)	15/31 (48.4)	-	-	-
Chloramphenicol	4/17 (23.5)	1/10 (10)	5/27 (18.5)	1/7 (14.3)	1/6 (16.7)	2/13 (15.4)
Colistin	-	-	-	1/6 (16.7)	0/8 (0)	1/14 (7.1)
Levofloxacin	1/12 (8.3)	5/12 (41.7)	6/24 (25)	2/3 (66.7)	1/4 (25)	3/7 (42.9)
Norfloxacin	-	1/1 (100)	1/1 (100)	0/1 (0)	-	0/1 (0)
Ofloxacin	2/5 (40)	0/3 (0)	2/8 (25)	-	1/1 (100)	1/1 (100)
Moxifloxacin	2/15 (13.3)	1/11 (9.1)	3/26 (11.5)	-	-	-
Doxycycline	4/14 (28.6)	3/12 (25)	7/26 (26.9)	0/1 (0)	0/2 (0)	0/3 (0)
Nitrofurantoin	0/2 (0)	1/3 (33.3)	1/5 (20)	-	-	-
Quinupristin	0/1 (0)	-	0/1 (0)	-	-	-
Linezolid	0/19 (0)	0/16 (0)	0/35 (0)	-	-	-
Erythromycin	9/17 (52.9)	8/13 (61.5)	17/30 (56.7)	-	-	-
Gentamycin	2/8 (25)	5/14 (35.7)	7/22 (31.8)	2/7 (28.6)	4/9 (44.4)	6/16 (37.5)
Ertapenem	0/1 (0)	0/1 (0)	0/2 (0)	0/4 (0)	2/3 (66.7)	2/7 (28.6)
Imipenem	0/1 (0)	-	0/1 (0)	0/6 (0)	5/10 (50)	5/16 (31.25)
Meropenem	0/1 (0)	0/1 (0)	0/2 (0)	0/8 (0)	7/10 (70)	7/18 (38.9)
Oxacillin	12/18 (66.7)	8/13 (61.5)	20/31 (64.5)	-	-	-
Penicillin	15/21 (71.4)	16/18 (88.9)	31/39 (79.5)	-	-	-
Piperacillin	1/1 (100)	1/1 (100)	2/2 (100)	-	2/2 (100)	2/2 (100)
Rifampicin	3/14 (21.4)	3/11 (27.3)	6/25 (24)	-	-	-
TMP+SMTX	6/14 (42.9)	5/12 (41.7)	11/26 (42.3)	2/6 (33.3)	6/10 (60)	8/16 (50)
Piperacillin + Tazobactam	0/1 (0)	-	0/1 (0)	0/5 (0)	6/9 (66.7)	6/14 (42.9)
Teicoplanin	1/2 (50)	0/2 (0)	1/4 (25)	-	-	-
Tetracycline	7/16 (43.75)	5/15 (33.3)	12/31 (38.7)	3/5 (60)	2/7 (28.6)	5/12 (41.7)
Tigecycline	1/10 (10)	2/8 (25)	3/18 (16.7)	1/5 (20)	1/6 (16.7)	2/11 (18.2)
Tobramycin	1/1 (100)	-	1/1 (100)	1/3 (33.3)	4/7 (57.1)	5/10 (50)
Vancomycin	0/6 (0)	0/7 (0)	0/13 (0)	-	-	-

Non-N= non-nosocomial, N=Nosocomial, , TMP+SMTX=Trimethoprim with sulphamethoxazole

Table S3 *In vitro* resistance to antibiotics for *Staphylococcus aureus* ascitic infection

Staphylococcus aureus	Non-N Resistance/Total (%)	N Resistance/Total (%)	TOTAL Resistance/Total (%)
Amikacin	3/8 (37.5)	3/4 (75)	6/12 (50)
Ampicillin	0/1 (0)	-	0/1 (0)
Ceftaroline	1/2 (50)	2/3 (66.7)	3/5 (60)
Ciprofloxacin	3/13 (23.1)	5/12 (41.7)	8/25 (32)
Clarithromycin	7/13 (53.8)	7/11 (63.6)	14/24 (58.3)
Clindamycin	8/13 (61.5)	7/12 (58.3)	15/25 (60)
Chloramphenicol	4/13 (30.8)	1/9 (11.1)	5/22 (22.7)
Levofloxacin	0/6 (0)	5/10 (50)	5/16 (31.2)
Moxifloxacin	2/12 (16.7)	1/10 (10)	3/22 (13.6)
Doxycycline	3/12 (25)	2/11 (18.2)	5/23 (21.7)
Quinupristin	0/1 (0)	-	0/1 (0)
Linezolid	0/12 (0)	0/11 (0)	0/23 (0)
Erythromycin	9/13 (69.2)	7/12 (58.3)	16/25 (64)
Gentamycin	2/5 (40)	3/10 (30)	5/15 (33.3)
Oxacillin	7/13 (53.8)	6/11 (54.5)	13/24 (54.2)
Penicillin	13/13 (100)	12/12 (100)	25/25 (100)
Piperacillin	-	1/1 (100)	1/1 (100)
Rifampicin	3/12 (25)	3/11 (27.3)	6/23 (26.1)
Trimethoprim+Sulfamethoxazole	4/12 (33.3)	4/11 (36.4)	8/23 (34.8)
Teicoplanin	0/1 (0)	-	0/1 (0)
Tetracycline	4/10 (40)	4/10 (40)	8/20 (40)
Tigecycline	1/8 (12.5)	2/8 (25)	3/16 (18.7)
Tobramycin	1/1 (100)	-	1/1 (100)
Vancomycin	-	0/1 (0)	0/1 (0)

Non-N= non-nosocomial, N=Nosocomial

Table S4 *In vitro* resistance to antibiotics for *Streptococcus spp.* ascitic infection

Streptococcus spp.	Non-N Resistance/Total (%)	N Resistance/Total (%)	TOTAL Resistance/Total (%)
Ampicillin	1/3 (33.3)	-	1/3 (33.3)
Ampicillin + Clavulanic Acid	0/1 (0)	-	0/1 (0)
Cefepime	0/3 (0)	0/2 (0)	0/5 (0)
Cefotaxime	0/3 (0)	0/2 (0)	0/5 (0)
Cefoxitin	1/1 (100)	-	1/1 (100)
Ceftriaxone	1/2 (50)	0/2 (0)	1/4 (25)
Clarithromycin	0/3 (0)	0/1 (0)	0/4 (0)
Clindamycin	0/4 (0)	0/1 (0)	0/5 (0)
Chloramphenicol	0/3 (0)	0/1 (0)	0/4 (0)
Levofloxacin	1/3 (33.3)	-	1/3 (33.3)
Ofloxacin	2/3 (66.7)	0/1 (0)	2/4 (50)
Moxifloxacin	0/2 (0)	0/1 (0)	0/3 (0)
Doxycycline	1/1 (100)	1/1 (100)	2/2 (100)
Linezolid	0/3 (0)	0/1 (0)	0/4 (0)
Erythromycin	0/3 (0)	1/1 (100)	1/4 (25)
Ertapenem	0/1 (0)	0/1 (0)	0/2 (0)
Imipenem	0/1 (0)	-	0/1 (0)
Meropenem	0/1 (0)	0/1 (0)	0/2 (0)
Oxacillin	1/1 (100)	-	1/1 (100)
Penicillin	2/4 (50)	1/2 (50)	3/6 (50)
Piperacillin	1/1 (100)	-	1/1 (100)
Rifampicin	0/1 (0)	-	0/1 (0)
Trimethoprim + Sulfamethoxazole	1/1 (100)	1/1 (100)	2/2 (100)
Piperacillin + Tazobactam	0/1 (0)	-	0/1 (0)
Tetracycline	2/3 (66.7)	0/1 (0)	2/4 (50)
Tigecycline	0/1 (0)	-	0/1 (0)
Vancomycin	0/3 (0)	0/2 (0)	0/5 (0)

Non-N= non-nosocomial, N=Nosocomial

Table S5 *In vitro* resistance to antibiotics for *Enterococcus spp.* ascitic infection

Enterococcus spp.	Non-N Resistance/Total (%)	Nosocomial Resistance/Total (%)	TOTAL Resistance/Total (%)
Ampicillin	0/3 (0)	3/4 (75)	3/7 (42.9)
Ciprofloxacin	0/3 (0)	1/4 (25)	1/7 (14.3)
Levofloxacin	0/3 (0)	0/2 (0)	0/5 (0)
Norfloxacin	-	1/1 (100)	1/1 (100)
Ofloxacin	0/2 (0)	0/2 (0)	0/4 (0)
Doxycycline	0/1 (0)	-	0/1 (0)
Nitrofurantoin	0/2 (0)	1/3 (33.3)	1/5 (20)
Linezolid	0/3 (0)	0/4 (0)	0/7 (0)
Gentamycin	0/2 (0)	2/4 (50)	2/6 (33.3)
Oxacillin	3/3 (100)	2/2 (100)	5/5 (100)
Penicillin	0/3 (0)	3/4 (75)	3/7 (42.9)
Teicoplanin	1/1 (100)	0/2 (0)	1/3 (33.3)
Tetracycline	0/2 (0)	1/4 (25)	1/6 (16.7)
Tigecycline	0/1 (0)	-	0/1 (0)
Vancomycin	0/3 (0)	0/4 (0)	0/7 (0)

Non-N= non-nosocomial, N=Nosocomial

Table S6 *In vitro* resistance to antibiotics for *E. coli* ascitic infection			
E. coli	Non-N<br>Resistance/Total (%)	Nosocomial<br>Resistance/Total (%)	TOTAL<br>Resistance/Total (%)
Amikacin	-	0/2 (0)	0/2 (0)
Aztreonam	0/3 (0)	0/3 (0)	0/6 (0)
Ampicillin	3/3 (100)	2/2 (100)	5/5 (100)
Ampicillin + Clavulanic Acid	0/3 (0)	0/3 (0)	0/6 (0)
Ampicillin + Sulbactam	0/2 (0)	0/2 (0)	0/4 (0)
Cefazolin	1/3 (33.3)	0/1 (0)	1/4 (25)
Cefepime	0/3 (0)	0/3 (0)	0/6 (0)
Cefotaxime	0/2 (0)	-	0/2 (0)
Cefoxitin	0/3 (0)	0/2 (0)	0/5 (0)
Ceftazidime	0/3 (0)	0/3 (0)	0/6 (0)
Ceftazidime + Avibactam	-	0/1 (0)	0/1 (0)
Ceftriaxone	0/3 (0)	0/3 (0)	0/6 (0)
Cefuroxime	0/3 (0)	0/3 (0)	0/6 (0)
Ciprofloxacin	1/3 (33.3)	0/3 (0)	1/6 (16.7)
Chloramphenicol	0/3 (0)	0/2 (0)	0/5 (0)
Colistin	0/1 (0)	0/2 (0)	0/3 (0)
Levofloxacin	0/1 (0)	0/2 (0)	0/3 (0)
Gentamycin	1/2 (50)	0/3 (0)	1/5 (20)
Ertapenem	0/2 (0)	0/1 (0)	0/3 (0)
Imipenem	0/2 (0)	0/3 (0)	0/5 (0)
Meropenem	0/3 (0)	0/2 (0)	0/5 (0)
Trimethoprim + Sulfamethoxazole	1/2 (50)	1/3 (33.3)	2/5 (40)
Piperacillin + Tazobactam	0/2 (0)	0/2 (0)	0/4 (0)
Tetracycline	0/1 (0)	0/2 (0)	0/3 (0)
Tigecycline	0/2 (0)	0/2 (0)	0/4 (0)
Tobramycin	0/1 (0)	0/1 (0)	0/2 (0)

Non-N= non-nosocomial, N=Nosocomial

Table S7 *In vitro* resistance to antibiotics for *Klebsiella spp*. ascitic infection			
Klebsiella	Non-N Resistance/Total (%)	Nosocomial Resistance/Total (%)	TOTAL Resistance/Total (%)
Amikacin	0/1 (0)	1/3 (33.3)	1/4 (25)
Aztreonam	0/2 (0)	0/2 (0)	0/4 (0)
Ampicillin	1/1 (100)	1/1 (100)	2/2 (100)
Ampicillin + Clavulanic Acid	1/2 (50)	2/3 (66.7)	3/5 (60)
Ampicillin + Sulbactam	1/1 (100)	2/3 (66.7)	3/4 (75)
Cefazolin	1/1 (100)	3/3 (100)	4/4 (100)
Cefepime	0/2 (0)	2/2 (100)	2/4 (50)
Cefotaxime	-	3/3 (100)	3/3 (100)
Cefoxitin	0/2 (0)	3/3 (100)	3/5 (60)
Ceftazidime	0/1 (0)	3/3 (100)	3/4 (75)
Cefoperazone + Sulbactam	0/2 (0)	0/1 (0)	0/3 (0)
Ceftazidime + Avibactam	0/1 (0)	0/1 (0)	0/2 (0)
Ceftriaxone	0/2 (0)	3/3 (100)	3/5 (60)
Cefuroxime	0/1 (0)	3/3 (100)	3/4 (75)
Ciprofloxacin	0/2 (0)	3/3 (100)	3/5 (60)
Chloramphenicol	0/2 (0)	0/3 (0)	0/5 (0)
Colistin	0/2 (0)	0/2 (0)	0/4 (0)
Gentamycin	0/1 (0)	1/2 (50)	1/3 (33.3)
Ertapenem	0/2 (0)	2/2 (100)	2/4 (50)
Imipenem	0/1 (0)	2/3 (66.7)	2/4 (50)
Meropenem	0/2 (0)	2/2 (100)	2/4 (50)
Trimethoprim + Sulfamethoxazole	0/2 (0)	2/3 (66.7)	2/5 (40)
Piperacillin + Tazobactam	-	1/1 (100)	1/1 (100)
Tetracycline	1/1 (100)	1/3 (33.3)	2/4 (50)
Tigecycline	0/1 (0)	0/2 (0)	0/3 (0)
Tobramycin	-	2/2 (100)	2/2 (100)

Non-N= non-nosocomial, N=Nosocomial

Table S8 In vitro resistance to antibiotics for *Acinetobacter baumanii* ascitic infection			
Acinetobacter baumanii	Non-N Resistance/Total (%)	Nosocomial Resistance/Total (%)	TOTAL Resistance/Total (%)
Amikacin	0/1 (0)	2/2 (100)	2/3 (66.7)
Ampicillin + Sulbactam	1/1 (100)	2/2 (100)	3/3 (100)
Cefazolin	-	1/1 (100)	1/1 (100)
Cefepime	1/1 (100)	2/2 (100)	3/3 (100)
Cefotaxime	1/1 (100)	3/3 (100)	4/4 (100)
Ceftazidime	1/1 (100)	3/3 (100)	4/4 (100)
Cefoperazone + Sulbactam	-	1/1 (100)	1/1 (100)
Ceftaroline	-	1/1 (100)	1/1 (100)
Ceftriaxone	1/1 (100)	1/1 (100)	2/2 (100)
Ciprofloxacin	1/1 (100)	3/3 (100)	4/4 (100)
Colistin	0/1 (0)	0/2 (0)	0/3 (0)
Levofloxacin	1/1 (100)	1/1 (100)	2/2 (100)
Doxycycline	0/1 (0)	0/2 (0)	0/3 (0)
Gentamycin	0/1 (0)	2/2 (100)	2/3 (66.7)
Imipenem	0/1 (0)	2/2 (100)	2/3 (66.7)
Meropenem	0/1 (0)	3/3 (100)	3/4 (75)
Piperacillin	-	1/1 (100)	1/1 (100)
Trimethoprim + Sulfamethoxazole	0/1 (0)	2/3 (66.7)	2/4 (50)
Piperacillin + Tazobactam	0/1 (0)	3/3 (100)	3/4 (75)
Tetracycline	1/1 (100)	1/1 (100)	2/2 (100)
Tigecycline	0/1 (0)	0/1 (0)	0/2 (0)
Tobramycin	1/1 (100)	1/2 (50)	2/3 (66.7)

Non-N= non-nosocomial, N=Nosocomial
